# Prevalence and Determinants of Depressive Symptoms among Young Adolescents in Malaysia: A Cross-Sectional Study

**DOI:** 10.3390/children10010141

**Published:** 2023-01-11

**Authors:** Sarbhan Singh, Chee Herng Lai, Nuur Hafizah Md Iderus, Sumarni Mohd Ghazali, Lonny Chen Rong Qi Ahmad, Lim Mei Cheng, Mohamad Nadzmi Nadzri, Asrul Anuar Zulkifli, Jeyanthi Suppiah, Rafdzah Ahmad Zaki, Nik Daliana Nik Farid, Rama Krishna Supramanian, Awatef Amer Nordin, Chong Zhuo Lin, Kushilpal Kaur, Nur’ain Mohd Ghazali

**Affiliations:** 1 Institute for Medical Research (IMR), National Institutes of Health (NIH), Ministry of Health Malaysia, Setia Alam 40170, Malaysia; 2 Department of Social and Preventive Medicine, Faculty of Medicine, University of Malaya, Kuala Lumpur 50603, Malaysia; 3 Institute for Health System Research (IHSR), National Institutes of Health (NIH), Ministry of Health Malaysia, Setia Alam 40170, Malaysia; 4 Institute for Public Health (IPH), National Institutes of Health (NIH), Ministry of Health Malaysia, Setia Alam 40170, Malaysia; 5 Department of Psychiatry, Hospital Selayang, Ministry of Health, Malaysia, Batu Caves 68100, Malaysia

**Keywords:** depressive symptoms, adolescents, determinants, Malaysia

## Abstract

Depression is the most common mental health problem affecting adolescents globally, wherein its increasing prevalence together with the negative health impacts escalates the need for further research in this area. This work determined the prevalence and factors associated with depressive symptoms among young adolescents in Malaysia. A total of 1350 adolescent aged 13 to 14 years in school across nine secondary schools in Selangor state, Malaysia participated in a cross-sectional study. Independent variables were examined using the using the Global School-Based Student Health Survey included age, gender, ethnicity, alcohol intake, smoking and illicit drug use, loneliness, bullying, parental marital status, income and supervision; and the Health Literacy and Stigma questionnaire examined mental health literacy levels. Depressive symptoms were the dependent variable which was examined using the Center for Epidemiology Study Depression (CESD) instrument. Prevalence of depressive symptoms among all participants was 19 % (95% CI [16.9, 21.2]), with a higher prevalence of depressive symptoms being reported among females 26.3% (95% CI [23.0, 29.8]) compared to males 11.7% (95% CI [9.4, 14.4]). Determinants namely females (*AOR* = 3.83; 95% CI [2.66, 5.52]), smoking (*AOR* = 6.16; 95% CI [3.15, 12.05]), been bullied (*AOR* = 3.70; 95% CI [2.51, 5.47]), felt lonely (*AOR* = 10.46; 95% CI [7.09, 15.42]) and having no parental supervision (*AOR* = 1.79; 95% CI [1.26, 2.53]) significantly increased the odds of depressive symptoms among all adolescents in the multivariate model. In addition, feeling lonely, being bullied and smoking were identified as common significant determinants of depressive symptoms across both genders. Feeling lonely (65% to 71%) and being bullied (10% to 19%) were ranked as the most important determinants of depressive symptoms among young adolescents. Tackling these factors would be instrumental in helping decision makers formulate depression prevention strategies and activities for adolescents.

## 1. Introduction

Individuals between the ages of 10 to 19 years are identified as adolescents, wherein they are classified as early and late adolescents, in all they make up a quarter of the global population [1,2]. Individuals in adolescence begin to explore their identities and positions in society, which makes this phase a critical phase between the progression into adulthood from childhood. This period is considered to have more biopsychosocial changes than other life stages, apart from infancy [3]. As a result, these developmental processes and changes may be associated with high stress levels and emotional instabilities [4]. In 2021, the World Health Organization (WHO) reported that globally about 10 to 20% of adolescents or one in seven adolescents suffer from at least one type of mental disorder, and therefore resulting in 13% of the total global disease burden affecting adolescents [5,6]. In addition, suicide and depression have been reported as significant contributors to mortality and morbidity among adolescents [5,6]. Evidence in literature suggests that depression is the most common mental disorder affecting adolescents, and its prevalence is on the rise [7,8].

The vast majority of studies on adolescent depression have been centered around the United States (US) and European regions which are high income nations [7,8,9], while fewer studies were conducted in low- and middle-income countries (LMICs) [10]. This is of concern, as 80% of depression burden occurs among people living in LMICs. Furthermore, depressive symptoms are highly prevalent among adolescents residing in LMICs in the Middle East, Africa, and Asia regions [9]. The prevalence of depressive symptoms among adolescents in the Southeast Asian (SEA) region have been reported at 34.9% (2012) in Thailand [11], 33.1% (2019) in Malaysia [12], 32.9% (2019) in Laos [13], 29.3%, (2021) in Indonesia [14], and 27.2% (2016) in Myanmar [15]. In Malaysia, previous research conducted have found an increased prevalence of depressive symptoms among adolescents from 17.7% to 33.1% from the year 2014 to 2019 [12,16]. These findings pose a threat to the mental and physical wellbeing of adolescents as depression would result in psychosocial impairment, frequent hospitalizations and risky behaviors such as drug and alcohol abuse [17].

The rising prevalence of depressive symptoms among adolescents has driven many researchers to identify the determinants or factors associated with depression among this population group so that measures can be taken early on to prevent depression before a formal diagnosis is made. As a result, the current available literature reports several determinants which could escalate the risk of depressive symptoms among adolescents. Among them include socio-demographic factors such as gender, age, educational attainment, parental marital and financial status, and high-risk behaviors (smoking, alcohol consumption and drug use) [11,12,14,16]. In addition to these factors, more recent studies have also found strong relationship between adolescent depression and mental health literacy (MHL) [18,19].

However, in Malaysia, a large number of research focusing on the prevalence and determinants of depressive symptoms have been conducted among older adolescents perusing their tertiary education (universities) instead of school-going adolescents [20,21]. Furthermore, among depression studies involving school-going adolescents, the majority involved older adolescents [19,22]. One possible reason for this could be attributed to the lower mental disorder morbidity and mortality among younger adolescents [5,6]. However, this is of concern as almost half of mental health disorders occur prior to 14 years of age and tend to increase in severity with the advancement in age [23]. Moreover, studies report that the mean age for adolescent to develop mental disorders is around 15 years and symptoms commonly develop as early as 2 to 3 years prior to the diagnosis, suggesting that young adolescents may have already exhibited symptoms of mental disorders from as young as 12–13 years old [24]. In addition, early onset depression among young adolescent results in undesired disease outcomes, poor academic performance, social disturbances, repeated depressive period occurrence during adulthood and increased suicidal risk [25,26]. Therefore, it is important to examine the determinants of depressive symptoms among young adolescents which could potentially prevent depression early on. Moreover, there are limited studies that have specifically examined the determinants of depressive symptoms unique to each gender among young adolescents in Malaysia. This is an important aspect, as gender has been reported as a significant factor for depression, wherein the majority of studies reported higher risk of depression among females compared to their male counterparts [7,12]. Consequently, it is crucial to determine the factors of depressive symptoms exclusive to each gender group.

To address the above issues, this work determined the prevalence and factors associated with depressive symptoms among young adolescents in Malaysia. In addition, this work provides a rank order of the most important determinants for depressive symptoms among adolescents. We hypothesize several sociodemographic factors (i.e., gender, smoking, alcohol intake, loneliness) would be significantly associated with depression among adolescents. Evidence generated from this work would highlight the burden of depression among young adolescents and form important findings regarding the factors of depressive symptoms unique to each gender. This would subsequently help decision makers in generating evidence-driven decisions to formulate depression prevention strategies and activities for adolescents.

## 2. Methods

### 2.1. Study Design

A cross-sectional study was carried out among National Secondary Schools students in Selangor State, Malaysia.

### 2.2. Population and Sample

As the most developed state in Malaysia, Selangor has an area span of 7951 square kilometers. The estimated total population of Selangor State in 2021 was 6.56 million [27]. The state has nine districts with a total of 225 national secondary schools with around 360,000 students. Adolescents aged between 13 and 14 years enrolled in a national secondary school education in Selangor State were regarded as the population source. This study focused on young adolescents (13 to 14 years) due to first the increasing burden of the mental health problem among these individuals, where the severity rises as age increases [28]. In addition, younger adolescents aged 13 to 14 years will not be sitting for major examination; therefore, variations in schooling schedule would not interfere with their educational activities.

The G* power software version 3.1 using the z-test family with a priori power analysis for logistic regression was used to estimate the sample size. The sample size software was parameterized using a (a) Two tail curve with a binomial distribution; (b) Adjusted Odds Ratio (AOR) between bullying and depression at 1.79 (95% CI [1.60–1.99]) based on Kaur et al. (2014) [16]; (c) likelihood of depression among those not bullied and bullied set at 0.10 and 0.29, respectively [16]; (d) power and alpha set at 80% and 0.05%, respectively; and (e) additional exposure variables (R2) AOR set at zero to account for their effects. The sample was inflated by 25% to account for the non-response rate. The final sample size required for the study was 1350 (Supplementary S1). Using a two-stage stratified random sampling method, all 225 national secondary schools in Selangor were categorized based on the districts and one school was randomly sampled from each district, resulting in a total of 9 schools. Students attending lower-secondary schools were randomly selected to join the study based on the proportional allocation of study participants according to class size at the school level

### 2.3. Data Collection

This study was registered with the National Medical Research Register (NMRR-18-719-40569) and had obtained ethical approval from the University of Malaya Research Ethical Committee (UM.TNC 2/UMREC). Data collection was performed at the respective schools on selected dates and times provided by the schools. Prior to data collection, all participants were informed regarding the ethical aspects of the study including voluntary participation in the study, anonymity of data, withdrawal right at any time during the study and study objectives. Subsequently study information sheets and consent forms were provided to all participants for their reference as well as for them to share it with their parents/guardians for obtaining consent. A total of 1350 students consented to join the study. All students who consented to participate in the study were then given the study questionnaires which were completed in the respective schools. The study questionnaires were coded using serial numbers and had no personal identifiers; therefore, ensuring the participant’s confidentiality. The participants completed the questionnaires within 15 to 20 min. All questionnaires were then collected by the researcher upon completion and checked for incomplete data immediately after each session.

### 2.4. Measures

The Global School Based Student Health Survey was used to obtain information on sociodemographic characteristics such as age, gender, ethnicity, alcohol intake, illicit drug use, smoking, bullied, feeling lonely, parental marital status, supervision and income [29,30].

The Mental Health Literacy and Stigma questionnaire (MHLS) with a depressed vignette was used to determine the level of MHL among participants [31]. This instrument is a 3-point Likert scale-based questionnaire consisting of 90 items which assesses MHL knowledge, help seeking and stigma. The MHLS has been translated to several languages and have been reported to be a valid and reliable instrument for use among adolescents in Malaysia [31]. For this study the level of MHL was classified as adequate or inadequate [18].

The Center for Epidemiology Study Depression (CESD) instrument was utilized to assess depressive symptoms among participants. This instrument has 20 items in which responses are recorded on a 4-point Likert scale response. The CESD provides a continuous score that ranges from 0 to 60; subsequently, a cutoff point at 27 indicates the presence of depressive symptoms among adolescents in Malaysia [32,33]. The CESD has been translated to several languages and have been reported to be a valid and reliable instrument for use among adolescents in Malaysia [33,34]. Cronbach’s alpha (α = 0.88) was reported for the CESD in this study

### 2.5. Statistical Analysis

Data was analyzed using the Statistical Package for the Social Sciences (SPSS) version 26.0. Missing data, abnormal values and double entry were checked and performed prior to analysis. Frequency, percentages and 95% CI were computed for descriptive analysis. Sociodemographic differences among male and female participants were tested using the Chi-square/Fisher’s exact test wherein significant *p* values are indicative of significant difference in sociodemographic characteristics.

The determinants of depressive symptoms were examined using the multivariate binary logistic regression analysis. Prior to multivariate analysis, univariate analysis was performed wherein variables with *p* < 0.25 from the univariate analysis, were considered significant and entered into the multivariable analysis [35]. The enter method was used for the variable selection process in the multivariate analysis. The goodness of fit for the multivariate models developed was determine based on the (a) Omnibus test of model coefficient, wherein significant values indicate satisfactory goodness of fit and (b) Hosmer-Lemeshow goodness-of-fit test, wherein non-significant estimates indicate satisfactory goodness of fit. The multivariate model performance was assessed based on Nagelkerke R Square (r^2^) estimates and the overall model accuracy. *AOR*, 95% CI and significance were estimated to determine the degree of association and statistical significance in the multivariate binary logistic regression analysis and presented in tabular form for each model. The rank order of the most important variables in the multivariate models were determined based on an increment in the r^2^ estimates attributed by each significant variable in the model which were presented visually as bar charts [36]. Evidence of multicollinearity and interaction were tested using Cramers and interaction function in SPSS, respectively, to sufficiently meet the assumption of logistic regression [35]. These assumptions were adequately met (Supplementary S2 and S3).

The assumptions of logistic regression that were assessed were: Multicollinearity which was examined using the Cramer’s V wherein any two variables with a Cramer’s V value of >0.3 are considered to have evidence of multicollinearity [35]. Evidence of interaction was examined using the test of the interaction function in SPSS, whereby a significant value (*p* < 0.05) indicates that interaction is present. There was no evidence of multicollinearity and interaction among the independent variables (Supplementary S2 and S3). The multivariate analysis was conducted for all participants and subsequently for males and female participants separately, resulting in three multivariate models.

## 3. Results

### 3.1. Characteristics of Participants

Totally 1350 students enrolled in this study, of which the gender distribution was similar at 676 (50.1%) and 674 (49.9%), males and females, respectively. There were 685 (50.7%) and 665 (49.3%) students aged 13 and 14 years, respectively. A large number of participants were of Malay ethnicity (*n* = 897, 66.4%). In total, there were 77 (5.7%) participants who smoke and 111 (8.2%) participants who consume alcohol. Having been bullied and felt lonely were reported at 249 (18.4%) and 534 (39.6%), respectively. The majority of the participants have intact families (married and living together) (*n* = 1199, 88.8%) with moderate to high parental income level (*n* = 850, 63.0%) and were under parental supervision (*n* = 904, 67.0%). With regard to the level of MHL, the majority of participants were classified as having inadequate MHL (*n* = 1299, 96.2%). The prevalence of depressive symptoms among all participants was 19% (95% CI [16.9, 21.2]), with a higher prevalence of depressive symptoms being reported among females 26.3% (95% CI [23.0, 29.8]) compared to males 11.7% (95%CI 9.4, 14.4). No significant differences in baseline characteristics across both genders were reported, except for depressive symptoms, as shown in Table 1.

### 3.2. Determinants of Depressive Symptoms

#### 3.2.1. Model 1. All Participants

**Figure 1:**
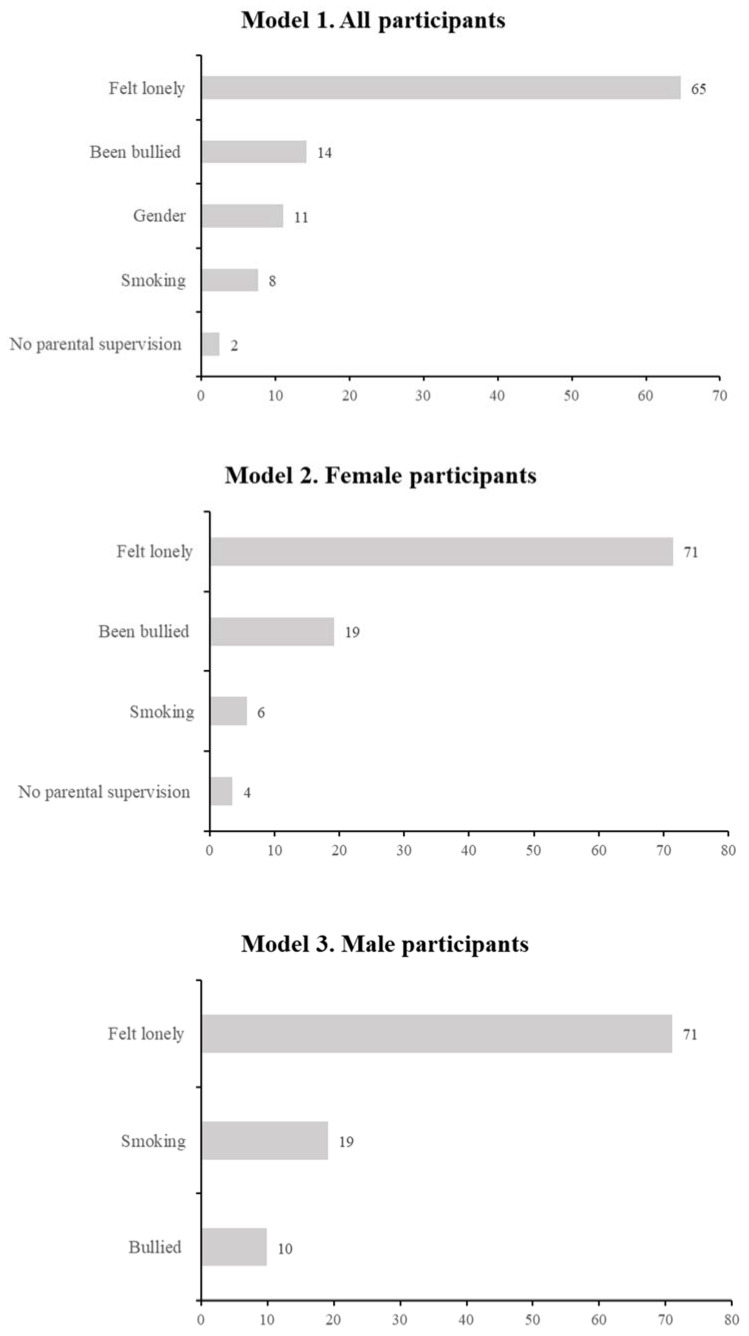
Rank order of important variables based on percentage (%) increase in r square, for each model.

In the bivariate logistic regression analysis, 11 variables namely age, gender, ethnicity, smoking, alcohol consumption, illicit drug use, been bullied, felt lonely, parental supervision, parental marital status and adequate MHL were reported to be significantly associated with depressive symptoms among all participants. However, in the final adjusted multivariate logistic regression model, only gender, smoking, been bullied, felt lonely and parental supervision showed statistical significance with depressive symptoms among all participants, with an overall model accuracy of 85.9%. Wherein determinants such as female gender (*AOR* = 3.83; 95% CI [2.66, 5.52]), smoking (*AOR* = 6.16; 95% CI [3.15, 12.05]), been bullied (*AOR* = 3.70; 95% CI [2.51, 5.47]), felt lonely (*AOR* = 10.46; 95% CI [7.09, 15.42]) and having no parental supervision (*AOR* = 1.79; 95% CI [1.26, 2.53]) significantly increased the odds of depressive symptoms among all participants, as shown in Table 2. The determinant with the highest importance in the model was felt lonely (65%), followed by been bullied (14%), gender (11%), smoking (8%) and no parental supervision (2%), as shown in Figure 1.

#### 3.2.2. Model 2. Female Participants

In the bivariate logistic regression analysis, nine variables namely age, ethnicity, smoking, alcohol consumption, illicit drug use, been bullied, felt lonely, parental supervision and adequate MHL were found to be statistically significantly associated with depressive symptoms among female participants. However, in the final adjusted multivariate logistic regression model, only smoking, been bullied, felt lonely and parental supervision showed statistical significance with depressive symptoms among female participants, with an overall model accuracy of 73.7%. Wherein factors such as smoking (*AOR* = 11.67; 95% CI [3.18, 22.85]), been bullied (*AOR* = 5.44; 95% CI [3.06, 9.67]), felt lonely (*AOR* = 12.06; 95% CI [7.24, 20.08]) and having no parental supervision (*AOR* = 2.23; 95% CI [1.41, 3.52]) significantly increased the odds of depressive symptoms among female participants, as shown in Table 3. The determinant with the highest importance in the model was felt lonely (71%), followed by been bullied (19%), smoking (6%) and no parental supervision (4%), as shown in Figure 1.

#### 3.2.3. Model 3. Male Participants

In the bivariate logistic regression analysis, seven variables namely smoking, alcohol consumption, illicit drug use, been bullied, felt lonely, parental income and parental supervision were found to be statistically significantly associated with depressive symptoms among male participants. However, in the final adjusted multivariate logistic regression model, only smoking, been bullied and felt lonely showed statistical significance with depressive symptoms among male participants, with an overall model accuracy of 88.0%. Wherein factors such as smoking (*AOR* = 4.64; 95% CI [2.05, 10.51]), being bullied (*AOR* = 2.56; 95% CI [1.45, 4.51]) and felt lonely (*AOR* = 8.15; 95% CI [4.37, 15.20]) significantly increased the odds of depressive symptoms among male participants, as shown in Table 4. The determinant with the highest importance in the model was felt lonely (71%), followed by smoking (19%), and been bullied (10%), as shown in Figure 1.

## 4. Discussion

This study determined the prevalence, sociodemographic and MHL determinants of depressive symptoms among young Malaysian adolescents. In addition, the significant determinants of depressive symptoms identified across the various models in this study were ranked in order of importance based on their effect towards depressive symptoms.

The overall prevalence of depressive symptoms in this study was reported at 19.0% (95% CI [16.9, 21.2]), which was higher than previous findings in 2007, 2014 and 2021 when the prevalence of depressive symptoms among secondary school-going adolescents in Malaysia was reported at 10.3%, 17.7% and 16.6%, respectively [37,38,39], but lower compared to findings reported by other local studies in 2016 and 2019 at 25.6% and 33.1%, respectively [12,33]. The prevalence of depressive symptoms in this study is lower compared to previous research conducted in neighboring SEA countries such as Thailand, Laos, Indonesia and Myanmar which had their reported prevalence of depressive symptoms among adolescents at 34.9%, 32.9%, 29.3% and 27.2%, respectively [11,13,14,15]. However, the findings in this study are higher compared to studies conducted in the United States of America (US) and European nations which had a reported prevalence of depressive symptoms among adolescents ranging from 7.1 to 18.0% [40,41]. Variation in the prevalence of depressive symptoms among adolescents across studies can be attributed to methodological variations, sample characteristics, study instruments, study locations and cultural differences.

This study also reported a higher prevalence of depressive symptoms among females 26.3% (95% CI [23.0, 29.8]) compared to males 11.7% (95% CI [9.4, 14.4]). Our findings are in line with previous research performed in the US, Chile and Canada [42,43,44]. Depressive symptoms are more prevalent among females due to genetic composition, elevated anxiety disorders, puberty, negative perceptions and sociocultural factors [42,43,44]. In addition, there are several hypotheses and theories that explain this gender difference, including the Gender Intensification hypothesis which indicates that depressive symptoms are more associated with feminine roles or stereotypes and the Stressful Life Events theory that suggests females are more vulnerable to psychological problems than males during adolescence, as they are subjected to more stressful life events [45,46].

In the multivariate logistic regression analysis involving all adolescents (model 1), gender, feeling lonely, being bullied, smoking and no parental supervision were found to be significantly associated with depressive symptoms in this study. More specifically this study found that females have significantly higher odds (*AOR* = 3.83, 95% CI [2.66, 5.52]) of developing depressive symptoms compared to their male counterparts. Our findings echo that of previous studies conducted in the US, Chile and Canada [42,44,47]. The reasons that could mediate this finding have been discussed above. Studies have consistently reported strong associations between loneliness and mental health problems among adolescents [10,48]. Loneliness can affect individuals at any time in life, and for some it can be an overwhelming feeling which ultimately may lead to negative thoughts and feelings. A longitudinal study conducted in Canada found that loneliness is considered a social risk factor for the escalation of depressive symptoms in early adolescence [49]. This is because loneliness which may occur as a result of poor experiences with peers or family members could result in social isolation, perceived low social acceptance, and poor self-esteem which is expected to underlie depressive symptoms [49]. In line with this evidence, the current study found that adolescents who feel lonely have 10.46 (95% CI [7.09, 15.42]) odds of developing depressive symptoms. Similar findings have been reported in studies conducted in China, Brazil and Australia [50,51,52].

Being bullied was also reported in this study as a significant determinant that could increase the odds of depressive symptoms among young adolescents by 3.70 times (95% CI [2.51, 5.47]). Previous research in Ethiopia, Denmark and India also reported similar findings [10,53,54]. The growing body of research suggests that bullying is a social phenomenon which is highest among young adolescents and is a form of an adverse life event that could have negative consequences on mental health [10,53,54]. This is because bullying results in reduced self-esteem and reduced desire to engage in social relations which could impair neurobiological and behavioral development, ultimately resulting in mental disorders [10,53,54]. In addition to gender, loneliness and bullying, this study also reports smoking as a significant factor that could increase the odds of depressive symptoms among adolescents by 6.16 times (95% CI [3.15, 12.05]). The findings of this study echo that of previous research in Finland, the US and United Kingdom [55,56,57]. The relationship between smoking and depression has been examined through complex models that consider direct effects, indirect and mediated effects. Smoking is considered as a negative form of a coping mechanism wherein individuals who indulge in it may be doing so in an effort to self-medicate an underlying problem or conform to social pressure which would ultimately result in a poor mental health outcome [58]. Furthermore, this study also reports that adolescents who have no parental supervision tended to have an increased risk of 1.79 (95% CI [1.26, 2.53]) of developing depressive symptoms. These findings are in line with studies conducted in Ethiopia and India [59,60]. A lack of parental supervision reflects a poor parent–child emotional connection which means that adolescents might not disclose their problems to their parents, which would delay seeking help. Furthermore, a greater sense of freedom resulting from a lack of parental supervision might encourage adolescents to be involved in high-risk behaviors which increases the odds of depressive symptoms [54].

This study also found that feeling lonely, being bullied and smoking were common determinants of depression for both male and female adolescents. In addition, feeling lonely was ranked as the most important variable as it accounted for 65% to 71% of the variance in depressive symptoms among adolescents, followed by bullying at 10% to 19%, across all three models. While our findings echoed previous studies, there is no universal consensus regarding the most important predictor for depressive symptoms among adolescents [61]. Traditionally, gender, race, health status and loneliness have been reported as important predictors of depressive symptoms [62].

The main strength in this study was its focus towards determining the factors associated with depressive symptoms among young adolescents accounting for the gender effect, which remains an important area to focus on. In addition, the randomly sampled participants from multiple schools across different districts would increase the generalization of the study findings in the local context. There are several limitations of the current study. First, being a cross-sectional study, it is challenging to establish a time–temporal relationship between predictors and outcome variables. Second, the ranking order of variables of importance employed in this study was based on the contribution of each variable towards improving the model ability to account for the variance of the outcome variables and therefore its interpretation must be performed with caution as there are various interpretations used in the literature when defining the importance of predictor variables. Third, the study focused on adolescents in Selangor state; therefore, generalization of findings to other states in Malaysia must be performed with caution.

## 5. Conclusions

This study has shown that the overall burden of depressive symptoms among young adolescents is on the rise, wherein females tend to suffer two-folds more depressive symptoms than males. Gender, feeling lonely, being bullied, smoking and no parental supervision remain significant determinants for depressive symptoms among young adolescents. In addition, feeling lonely, being bullied and smoking were identified as common significant determinants of depressive symptoms across both genders. Furthermore, feeling lonely and being bullied were ranked as the most important determinants of depressive symptoms among young adolescents. Therefore, to prevent and minimize the rising prevalence of depression among adolescents, it is pivotal that mental health policies urgently address these determinants of depression, by reviewing the existing school based mental health programs and campaigns to ensure that they are continuously being instituted, monitored and more importantly are aligned towards addressing factors such as loneliness, bullying, smoking and parental supervision. In addition, it would be important to educate and sensitize parents and teachers regarding these important determinants of depression as they could function as gate keepers that would promote early help seeking. Addressing these determinants of depression could be crucial in developing a younger and healthier generation of adolescents which would be able to contribute positively towards the community and nation.

## Figures and Tables

**Table 1:** Participants characteristics.

Characteristics		Total	Male	Female	Chi Square	*p*-Value
		(*N* = 1350)	(*n* = 676)	(*n* = 674)		
Age	13 years	685 (50.7)	342 (50.6)	343 (50.9)	0.012	0.913
	14 years	665 (49.3)	334 (49.4)	331 (49.1)		
Ethnicity	Malay	897 (66.4)	433 (64.1)	464 (68.8)	3.472	0.065
	Non-Malay	453 (33.6)	243 (35.9)	210 (31.2)		
Smoking status	Smoker	77 (5.7)	40 (5.9)	37 (5.5)	0.115	0.815
	Non-Smoker	1273 (94.3)	636 (94.1)	637 (94.5)		
Consume alcohol	Yes	111 (8.2)	66 (9.8)	45 (6.7)	4.261	0.05
	No	1239 (91.8)	610 (90.2)	629 (93.3)		
Illicit drug use	Yes	22 (1.6)	12 (1.8)	10 (1.5)	0.179	0.83
	No	1328 (98.4)	664 (98.2)	664 (98.5)		
Been bullied	Yes	249 (18.4)	133 (19.7)	116 (17.2)	1.362	0.262
	No	1101 (81.6)	543 (80.3)	558 (82.8)		
Felt lonely	Yes	534 (39.6)	253 (37.4)	281 (41.7)	2.568	0.119
	No	816 (60.4)	423 (62.3)	393 (58.3)		
Parental marital status	Married and living together	1199 (88.8)	606 (89.6)	593 (88.0)	0.939	0.343
	Divorced/	151 (11.2)	70 (10.4)	81 (12.0)		
	Separated					
Parental income	Low income	500 (37.0)	233 (34.5)	266 (39.5)	3.619	0.063
	Moderate/	850 (63.0)	443 (65.5)	408 (60.5)		
	high income					
Parental supervision	Yes	904 (67.0)	445 (65.8)	459 (68.1)	0.788	0.386
	No	446 (33.0)	231 (34.2)	215 (31.9)		
Adequate MHL	Adequate	51 (3.8)	24 (3.6)	27 (4.0)	0.193	0.672
	Inadequate	1299 (96.2)	652 (96.4)	647 (96.0)		
Depressive symptoms	Absent	1094 (81.0)	597 (88.3)	497 (73.7)	46.65	<0.001 *
	Present	256 (19.0)	79 (11.7)	177 (26.3)		

Note. * Significant set at *p* < 0.05.

**Table 2:** Factors associated with depressive symptoms among adolescents (N = 1350).

Variable	Univariate Logistic Regression		Multivariate Logistic Regression	
	Crude OR (95% CI)	*p*-Value	Adjusted OR (95%CI)	*p*-Value
Gender				
Male	1		1	
Female	2.69 (2.01, 3.60)	<0.001 *	3.83 (2.66, 5.52)	<0.001 **
Age				
13 years	1		1	
14 years	0.81 (0.61, 1.06)	<0.123 *	0.75 (0.53, 1.06)	0.098
Ethnicity				
Malay	1		1	
Non-Malay	0.77 (0.57, 1.03)	0.081 *	0.88 (0.58, 1.33)	0.535
Smoking status				
Non-smoker	1		1	
Smoker	10.9 (6.61, 17.97)	<0.001 *	6.16 (3.15, 12.05)	<0.001 **
Consume alcohol				
No	1		1	
Yes	1.93 (1.25, 2.97)	0.003 *	1.14 (0.58, 2.26)	0.703
Illicit drug use				
No	1		1	
Yes	4.42 (1.90, 10.31)	0.001 *	1.89 (0.54, 6.64)	0.323
Been bullied				
No	1		1	
Yes	5.05 (3.73, 6.85)	<0.001 *	3.70 (2.51, 5.47)	<0.001 **
Felt lonely				
No	1		1	
Yes	12.74 (8.90, 18.23)	<0.001 *	10.46 (7.09, 15.42)	<0.001 **
Parental marital status				
Married and living together	1		1	
Divorce/Separated	1.39 (0.93, 2.08)	0.106 *	1.43 (0.86, 2.39)	0.173
Parental income				
Low	1			
Moderate/high	0.95 (0.72, 1.26)	0.733		
Parental supervision				
Yes	1		1	
No	2.87 (2.17, 3.79)	<0.001 *	1.79 (1.26, 2.53)	0.001 **
Adequate MHL				
Yes	1		1	
No	2.83 (1.01, 7.92)	0.048 *	2.96 (0.63, 13.93)	0.170

Note. 1 indicates a reference group; CI, Confidence interval; OR, Odds ratio; * Variables significant at 0.25 following univariate analysis with no evidence of multicollinearity and interaction were included to multivariate analysis. ** Significant set at *p* < 0.05 following multivariate analysis; Omnibus model coefficient *p* < 0.001; Hosmer–Lemeshow goodness-of-fit test chi-square = 6.7 (df = 8), *p* = 0.462. (Enter method used for variable selection).

**Table 3:** Factors associated with depressive symptoms among females (*n* = 674).

Variable	Univariate Logistic Regression		Multivariate Logistic Regression	
	Crude OR (95% CI)	*p*-Value	Adjusted OR (95%CI)	*p*-Value
Age				
13 years	1		1	
14 years	0.74 (0.52, 1.04)	<0.083 *	0.65 (0.41, 1.03)	0.064
Ethnicity				
Malay	1		1	
Non-Malay	0.66 (0.45, 0.97)	0.036 *	0.61 (0.35, 1.06)	0.077
Smoking status				
Non-smoker	1		1	
Smoker	28.25 (9.84, 31.05)	<0.001 *	11.67 (3.18, 22.85)	<0.001 **
Consume alcohol				
No	1		1	
Yes	2.18 (1.17, 4.04)	0.014 *	1.77 (0.62, 5.05)	0.284
Illicit drug use				
No	1		1	
Yes	4.33 (1.21, 15.51)	0.025 *	0.47 (0.03, 7.06)	0.588
Been bullied				
No	1		1	
Yes	8.60 (5.54, 13.34)	<0.001 *	5.44 (3.06, 9.67)	<0.001 **
Felt lonely				
No	1		1	
Yes	15.52 (9.84, 24.48)	<0.001 *	12.06 (7.24, 20.08)	<0.001 **
Parental marital status				
Married and living together	1			
Divorce/Separated	1.30 (0.78, 2.15)	0.317		
Parental income				
Low	1			
Moderate/high	0.85 (0.60, 1.20)	0.357		
Parental supervision				
Yes	1		1	
No	3.89 (2.71, 5.59)	<0.001 *	2.23 (1.41, 3.52)	0.001 **
Adequate MHL				
Yes	1		1	
No	2.01 (0.72, 6.16)	0.177 *	2.48 (0.32, 19.45)	0.387

Note. 1 indicates a reference group; CI, Confidence interval; OR, Odds ratio; * Variables significant at 0.25 following univariate analysis with no evidence of multicollinearity and interaction were included to multivariate analysis. ** Significant set at *p* < 0.05 following multivariate analysis; Omnibus model coefficient *p* < 0.001; Hosmer–Lemeshow goodness-of-fit test chi-square = 4.9 (df = 8), *p* = 0.759. (Enter method used for variable selection).

**Table 4:** Factors associated with depressive symptoms among males (*n* = 676).

Variable	Univariate Logistic Regression		Multivariate Logistic Regression	
	Crude OR (95% CI)	*p*-Value	Adjusted OR (95%CI)	*p*-Value
Age				
13 years	1			
14 years	0.94 (0.59, 1.51)	<0.805		
Ethnicity				
Malay	1			
Non-Malay	1.10 (0.68, 1.79)	0.689		
Smoking status				
Non-smoker	1		1	
Smoker	8.69 (4.42, 17.06)	<0.001 *	4.64 (2.05, 10.51)	<0.001 **
Consume alcohol				
No	1		1	
Yes	2.26 (1.19, 4.30)	0.013 *	1.02 (0.46, 2.27)	0.960
Illicit drug use				
No	1		1	
Yes	5.70 (1.76, 18.40)	0.004 *	2.91 (0.65, 13.03)	0.162
Been bullied				
No	1		1	
Yes	3.57 (2.17, 5.86)	<0.001 *	2.56 (1.45, 4.51)	0.001 **
Felt lonely				
No	1		1	
Yes	10.10 (5.53, 18.46)	<0.001 *	8.15 (4.37, 15.20)	<0.001 **
Parental marital status				
Married and living together	1			
Divorce/Separated	1.48 (0.74, 2.95)	0.270		
Parental income				
Low	1		1	
Moderate/high	1.42 (0.84, 2.38)	0.189 *	1.11 (0.62, 2.00)	0.729
Parental supervision				
Yes	1		1	
No	2.06 (1.28, 3.30)	<0.003 *	1.39 (0.80, 2.40)	0.242
Adequate MHL				
Yes	1			
No	2.99 (0.90, 7.92)	0.998		

Note. 1 indicates a reference group; CI, Confidence interval; OR, Odds ratio; * Variables significant at 0.25 following univariate analysis with no evidence of multicollinearity and interaction were included to multivariate analysis. ** Significant set at *p* < 0.05 following multivariate analysis; Omnibus model coefficient *p* < 0.001; Hosmer–Lemeshow goodness-of-fit test chi-square = 3.3 (df = 8), *p* = 0.913. (Enter method used for variable selection).

## Data Availability

The data for this study is available upon request from the authors.

## Supplementary Materials

The following supporting information can be downloaded at: https://www.mdpi.com/article/10.3390/children10010141/s1, Supplementary S1: Sample size estimation using G Power. Supplementary S2: Test of multicollinearity; Supplementary S3: Test for interaction.
