# Supplementary material for: Prevalence and Determinants of Depressive Symptoms among Young Adolescents in Malaysia: A Cross-Sectional Study
Source: Children (Basel). 2023 Jan 11;10(1):141. doi: 10.3390/children10010141 (PMC9856963; doi:10.3390/children10010141)
Supplement: Supplementary file 1 [file children-10-00141-s001.zip › children-2102630-supplementary.pdf]

Supplementary materials

Supplementary S1. Sample size estimation using G Power.

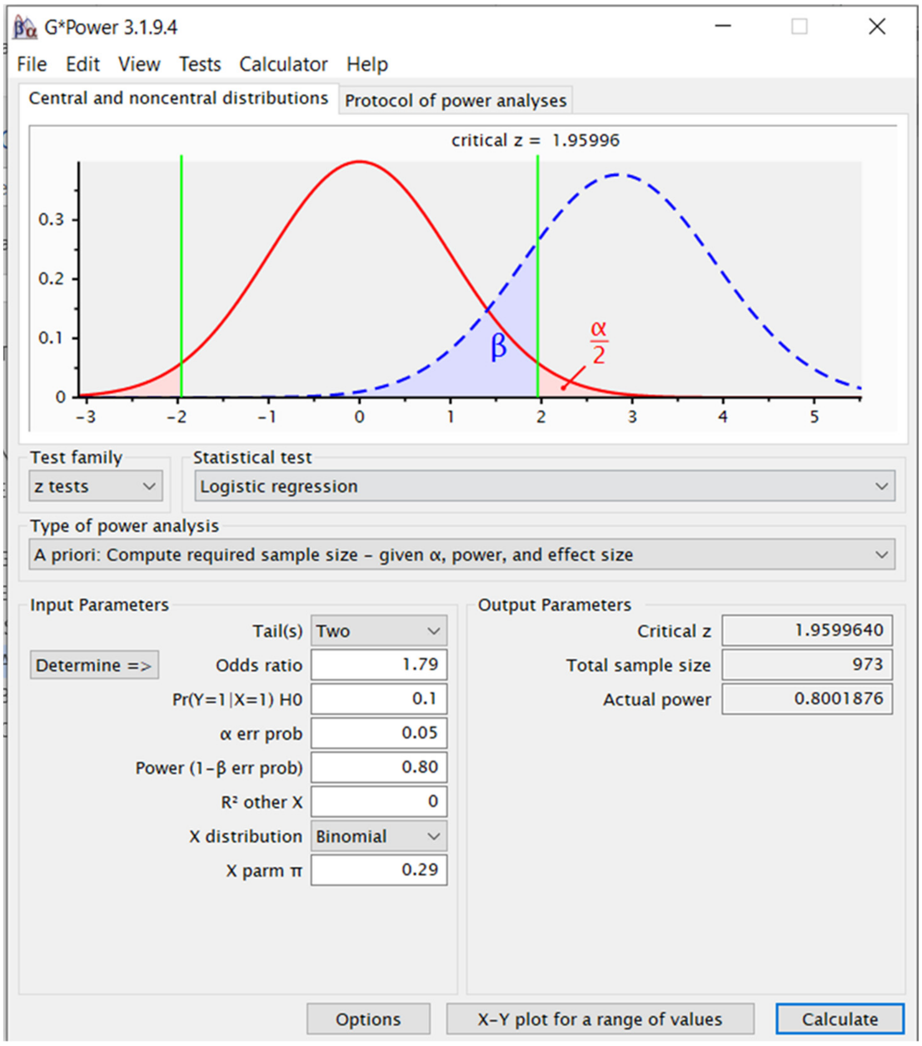

Supplementary S2. Test of multicollinearity.

Model 1. Variables in the Equation - Factors associated with depression among adolescents.

	Gende r	Age	Ethnicity	Smoke	Alcohol	Drug use	Bullied	Felt lonely	Parental marital status	Supervis ion	MHL
Gender	1.000	-.031	-.015	.125	.101	.043	.169	.089	.014	.049	.027
Age	-.031	1.000	.019	-.055	-.046	-.041	-.021	-.019	.029	.007	-.014
Ethnicity	-.015	.019	1.000	.054	-.019	-.137	.008	.050	.029	.088	.002
Smoke	.125	-.055	.054	1.000	-.164	-.149	-.051	.044	-.025	-.109	.115
Alcohol	.101	-.046	-.019	-.164	1.000	.024	-.042	-.072	-.005	.049	-.019
Drug use	.043	-.041	-.137	-.149	.024	1.000	-.008	.018	-.017	-.032	-.032
Bullied	.169	-.021	.008	-.051	-.042	-.008	1.000	.045	.016	-.056	.034
Felt lonely	.089	-.019	.050	.044	-.072	.018	.045	1.000	.062	-.053	-.053
Parental marital status	.014	.029	.029	-.025	-.005	-.017	.016	.062	1.000	.015	-.007
Supervision	.049	.007	.088	-.109	.049	-.032	-.056	-.053	.015	1.000	-.045
MHL	.027	-.014	.002	.115	-.019	-.032	.034	-.053	-.007	-.045	1.000

Note. Cramer's V values of more than 0.3 indicate evidence of multicollinearity among variables.

Model 2. Variables in the Equation - Factors associated with depression among female adolescent.

	Age	Ethnicity	Smoke	Alcohol	Drug use	Bullied	Felt lonely	Supervision	MHL
Age	1.000	.075	-.023	-.090	.041	-.059	-.045	-.009	-.006
Ethnicity	.075	1.000	.003	-.042	-.055	-.032	-.002	.043	-.026
Smoke	-.023	.003	1.000	-.028	-.203	-.111	.086	-.043	.194
Alcohol	-.090	-.042	-.028	1.000	-.130	-.003	-.008	.058	.038
Drug use	.041	-.055	-.203	-.130	1.000	-.066	.026	-.057	-.068
Bullied	-.059	-.032	-.111	-.003	-.066	1.000	.112	.010	.064
Felt lonely	-.045	-.002	.086	-.008	.026	.112	1.000	-.053	-.117
Supervision	-.009	.043	-.043	.058	-.057	.010	-.053	1.000	-.034
MHL	-.006	-.026	.194	.038	-.068	.064	-.117	-.034	1.000

Note. Cramer's V values of more than 0.3 indicate evidence of multicollinearity among variables.

Model 3. Variables in the Equation - Factors associated with depression among male adolescent.

	Smoke	Alcohol	Drug use	Bullied	Felt lonely	Parental monthly income	Supervision
Smoke	1.000	-.236	-.123	.000	-.003	.007	-.193
Alcohol	-.236	1.000	.015	-.072	-.105	-.027	.106
Drug use	-.123	.015	1.000	.017	.006	-.099	.011
Bullied	.000	-.072	.017	1.000	-.012	-.126	-.120
Felt lonely	-.003	-.105	.006	-.012	1.000	-.019	-.022
Parental monthly income	.007	-.027	-.099	-.126	-.019	1.000	.028
Supervision	-.193	.106	.011	-.120	-.022	.028	1.000

Note. Cramer's V values of more than 0.3 indicate evidence of multicollinearity among variables.

Supplementary S3. Test for interaction.

Model 1. Variables in the Equation - Factors associated with depression among adolescents.

		B	S.E.	Wald	df	Sig.	Exp(B)
Step 1a	Age by Alcohol by Bullied by Drug use by Ethnicity by Gender by Felt lonely by MHL by Parental marital status by Supervision by Smoke	9.408	2919.770	.000	1	.997	12191.313
	Constant	-1.472	.070	442.455	1	.000	.229
a. Variable(s) entered on step 1: Age * Alcohol * Bullied * Drug use * Ethnicity * Gender * Felt lonely * MHL * Parental marital status * Supervision * Smoke .							

Model 2. Variables in the Equation - Factors associated with depression among female adolescents.

		B	S.E.	Wald	df	Sig.	Exp(B)
Step 1a	Age by Alcohol by Bullied by Drug use by Ethnicity by Felt lonely by MHL by Supervision by Smoke	2.298	499.844	.000	1	.996	9.953
	Constant	-1.103	.090	150.610	1	.000	.332
a. Variable(s) entered on step 1: Age * Alcohol * Bullied * Drug use * Ethnicity * Felt lonely * MHL * Supervision * Smoke .							

Model 3. Variables in the Equation - Factors associated with depression among male adolescents.

		B	S.E.	Wald	df	Sig.	Exp(B)
Step 1a	Alcohol by Bullied by Drug use by Felt lonely by Parental monthly income by Supervision by Smoke	.790	.288	7.506	1	.600	2.203
	Constant	-2.158	.127	287.942	1	.000	.116
a. Variable(s) entered on step 1: Alcohol * Bullied * Drug use * Felt lonely * Parental monthly income * Supervision * Smoke .							

*Note. *Non-significant value ($p > 0.05$) indicates no evidence of interaction.*
